# Postural control in top-level female volleyball players

**DOI:** 10.1186/s13102-020-00213-9

**Published:** 2020-10-20

**Authors:** Dorota Borzucka, Krzysztof Kręcisz, Zbigniew Rektor, Michał Kuczyński

**Affiliations:** 1 Faculty of Physical Education and Physiotherapy, Opole University of Technology, ul. Prószkowska 76, 45-758 Opole, Poland; 2 Faculty of Physiotherapy, University School of Physical Education in Wroclaw, Al. I.J. Paderewskiego 35, 51-612 Wrocław, Poland

**Keywords:** Volleyball, Postural control, Posturography, Center of Pressure

## Abstract

**Background:**

The aim of this study was to compare the postural control of the Poland national women’s volleyball team players with a control group of non-training young women. It was hypothesized that volleyball players use a specific balance control strategy due to the high motor requirements of their team sport.

**Methods:**

Static postural sway variables were measured in 31 athletes and 31 non-training women. Participants were standing on a force plate with eyes open, and their center of pressure signals were recorded for the 20s with the sampling rate of 20 Hz in the medial-lateral (ML) and anterior-posterior (AP) planes.

**Results:**

In both AP and ML planes, athletes had lower range and higher fractal dimension of the COP. They had also higher peak frequency than control group in the ML plane only. The remaining COP indices including variability, mean velocity and mean frequency did not display any intergroup differences.

**Conclusion:**

It can be assumed that due to the high motor requirements of their sport discipline Polish female volleyball players have developed a unique posture control. On the court they have to distribute their sensory resources optimally between balance control and actions resulting from the specifics of the volleyball game. There are no clearly defined criteria for optimal postural strategies for elite athletes, but they rather vary depending on a given sport. The results of our research confirm this claim.

**Trial registration:**

The tests were previously approved by the Bioethical Commission of the Chamber of Physicians in Opole. (Resolution No. 151/13.12.2007). This study adheres to the CONSORT guidelines.

## Background

The ability to maintain a stable standing position is regarded as the fundamental objective in most motor activities. Human postural stability depends on the availability of intact sensory information including proprioceptive, vestibular, and visual afferent signals, central integration of those signals, and motor execution which must take into account various internal and environmental constraints. Although the textbook definition of upright stance maintenance seems to unfold this complex process, researchers still face numerous problems related to the understanding of the actual function of modalities responsible for postural control, their dependence on age and pathologies, and performance in extreme situations, for example, experienced by athletes.

The study of postural control in athletes could provide some insight into the development of specific postural strategies required by a particular sport. Postural stability in athletes of different sports has been studied by many authors [1–5]. However, there have been few studies of postural stability in top-level players. This is mainly due to the limited accessibility of elite athletes, who usually follow a tight schedule of training camps and championship competitions. The best researched group of athletes have been rifle shooters, who consistently displayed reduced sway velocity [6, 7]. A significantly decreased sway amplitude was also found in elite soccer players [8], gymnasts [9, 10], golfers [11], and ice hockey players [12, 13]. Notably, it has been proposed that gymnasts developed a unique ability to adapt their postural control more rapidly to the perceptive transition [9]. Due to the specificity of the gameplay and dynamically changing situations on the playing field, the perceptive transition is even more demanding in team games.

Efficient postural stability control is the basis for many activities in volleyball. The ability to maintain a stable posture when in contact with the ball is very important for a more precise match performance. During the game, the volleyball player usually adopts the so-called ready position, i.e. positioning of the body that enables her to be physically prepared to react to an upcoming play [14]. Many points scored or defended in volleyball are the results of airborne actions. However, an equally important contribution to the final score is being made by less spectacular actions including passing, setting, and digging that require perfect anticipation and timing, which, in turn, rely on stable, optimally aligned body position [15]. In volleyball it is illegal to hold the ball, so the player can only take action for a fraction of a second. The gameplay in volleyball is very quick, and many players’ actions are based on anticipation. For this reason, volleyball coaches attach great importance to the development of players’ balance ability in their training plans. On the other hand researchers try to understand the mechanisms of postural control in players at different sporting levels and use this knowledge not only for the optimization of the volleyball training process, but for other purposes, e.g. among the elderly, rehabilitation, etc. Many studies have also demonstrated that balance training has a significant influence on injury prevention in different sports [16–18]. The elimination of injuries is crucial for the optimization of training process in any sport.

The aim of the present study was to compare the postural control of the Poland national women’s volleyball team players with a control group of non-training young women. We have previously studied second-league male volleyball players [14]. The present study aims to determine whether similar postural strategies can be observed in elite female volleyball players. It is hypothesized that volleyball players use a specific balance control strategy due to the high motor requirements of their team sport.

## Methods

### Participants

Thirty-one players of the Poland national women’s volleyball team (age 25.7 ± 7.6 year; height 184.0 ± 7.7 cm; weight 72.4 ± 7.2 kg) were examined. The training experience of volleyball players ranged from 9 to 23 years. The research was carried out at the training camp, 10 months after winning the second gold medal at the European Women’s Volleyball Championship (Croatia 2005). The first medal was won 2 years earlier (Turkey 2003).The research was conducted with the consent of the Polish Volleyball Association, training staff and players. The control group consisted of 31 young women (age 20.7 ± 1.6 year; body height 168.2 ± 4.9 cm; body weight 61.8 ± 8.3 kg). These were students of the Faculty of Physical Education and Physiotherapy, who did not undertake systematic physical activity.

Students responded to a short questionnaire and those whose exercise physical activity was less than five times a week and less than 150 min a week were included to the control group. All of the students described their health condition as very good and agreed to participate in the study. All subjects gave an informed written consent form approved by the Bioethics Committee of Opole Chamber of Physicians in Opole.

### Measurement system

Ground reaction forces were acquired with a custom-made force plate strain gage type, in which output signals were amplified and delivered to an IBM computer.

### Study design

The study protocol was the same as at [14]. Participants were measured on a force plate with eyes open, and their COP signals were recorded for the 20s with the sampling rate of 20 Hz in the medial-lateral (ML) and anterior-posterior (AP) planes. The participants were requested to stand barefoot, with their arms at sides.

### Parameters

On the basis of the recorded COP signals, several parameters describing different properties of the postural control system were computed and compared between both groups. This included the measures of the COP variability [19]: standard deviation (SD), range (RA), and mean velocity (MV), and the indices of postural performance and complexity: peak frequency (PF) of COP-COM (center of mass) correction signal, COP frequency (CF) based on normalized path COP length and fractal dimension (FD) which quantifies the degree to which COP time series fills the metric time-space. Specifically, the peak frequency was derived from parameters of the viscoelastic model of standing posture whose detailed definition is covered in [20], whereas the COP frequency is defined as:

$$ CF=\frac{MV}{2\cdotp \pi \cdotp SD} $$

and fractal dimension:

$$ FD=\frac{\log 10(400)}{\log 10\left(400\cdotp \frac{RA}{MV\cdotp 20}\right)} $$

where: 400 is the number of samples (20s times 20 Hz).

### Statistics

Due to low *p*-values values of Shapiro-Wilk tests of normality, all dependent variables were subjected to Mann-Whitney U group independent tests in the anterior-posterior and medio-lateral plane separately. The effect size statistic for the Mann–Whitney test is *r* is the *Z* value from the test divided by the total number of observations.: small 0.1- < 0.3, medium 0.3- < 0.5, large > = 05. Statistical evidence of significance was set at *p* < 0.05. All tests were conducted with free and open software JAMOVI, Version 1.2 (retrieved from https://www.jamovi.org) and in R with rcompanion [21].

## Results

The results of the experiment are presented in Table 1. In both AP and ML planes, athletes had lower range and higher fractal dimension of the COP. They had also higher COP – COM frequency than control group in the ML plane only. The remaining COP indices including variability, mean velocity and mean frequency did not display any intergroup differences.

**Table 1:** Median and quartiles of COP parameters for both groups and the corresponding Mann-Whitney U independent tests used to compare differences between students and athletes

	Group	N	Median	Quartiles	W statistic	p	r effect size
**AP**							
SD	students	31	3.64	2.90–4.29	365.0	0.105	−0.207
	athletes	31	3.12	2.63–3.78			
RA	students	31	20.18	16.05–23.83	199.5	**< .001**	−0.503
	athletes	31	15.51	12.81–17.03			
MV	students	31	6.71	5.29–8.99	450.0	0.673	−0.055
	athletes	31	6.51	5.96–7.63			
CF	students	31	0.30	0.22–0.40	377.0	0.150	0.177
	athletes	31	0.35	0.30–0.41			
FD	students	31	1.46	1.37–1.57	246.5	**0.001**	0.422
	athletes	31	1.56	1.52–1.64			
PF	students	31	0.56	0.52–0.67	407.0	0.304	0.131
	athletes	31	0.61	0.54–0.70			
**ML**							
SD	students	31	2.88	2.15–3.43	361.0	0.094	−0.213
	athletes	31	2.43	1.92–3.08			
RA	students	31	16.06	13.10–21-05	250.5	**0.001**	−0.413
	athletes	31	11.85	9.30–15.77			
MV	students	31	6.77	5.27–7.53	407.0	0.306	−0.131
	athletes	31	6.41	4.80–7.17			
CF	students	31	0.39	0.32–0.47	450.0	0.672	0.058
	athletes	31	0.40	0.36–0.43			
FD	students	31	1.52	1.46–1.59	248.5	**0.001**	0.419
	athletes	31	1.64	1.56–1.67			
PF	students	31	0.68	0.59–0.76	286.0	**0.006**	0.345
	athletes	31	0.78	0.67–0.90			

*SD* Standard deviation of COP, *RA* A range of COP, *MV* Mean speed of COP, *CF* COP frequency, *FD* Fractal dimension, *PF* Peak frequency of COP-COM, *AP* Anterior-posterior plane, *ML* Medial-lateral plane, *N* Number of participants, W statistic of Mann-Whitney U independent test, r effect size - *Z* value from the test divided by the total number of observations

## Discussion

The aim of the study was to compare the postural control of top Polish female volleyball players (two-time European champions) and healthy, non-training young women. Postural stability assessment was carried out using COP variability measures: standard deviation, range, and mean velocity. It is assumed that the higher the values of these measures, the worse postural performance [22–24]. Their lower values indicate greater comfort, i.e. lower involvement of the central nervous system in the process of maintaining balance, lower energy expenditure, optimal involvement of muscle parts and appropriate change in the contribution of these parts. The elite Polish female volleyball players exhibited lower COP range values than the control group, but there were no differences in the standard deviation values. This observation may indicate that although both measures are strongly correlated, and the standard deviation is considered a better indicator of COP signal variability as it is calculated from all the coordinates (from the whole process of maintaining balance), the COP range should not be abandoned too hastily. The COP range is characterized by two extreme points, i.e. it can display an unexpectedly high value if a random fluctuation occurs during the process of computerized stability assessment. The conclusion is that despite similar standard deviation values, the female volleyball players are much less vulnerable to accidental fluctuations and disturbances of postural control. Therefore, the postural control of high-class female volleyball players may be postulated as better and more reliable.

The COP mean velocity is obtained by dividing the total sway path by the respective time duration. Lower mean velocity values indicate less neuromuscular activity that is needed for muscular corrections, which accounts for better postural control including energetic demands and coordination abilities. Therefore it might be expected that mean velocity values in elite female volleyball players would be lower than in the control group. However, it turned out that there were no significant differences. Interestingly, in the case of second-league male volleyball players, the mean velocity was much higher than in non-training controls [14]. These discrepancies may be associated with the rate of development of specific postural strategies in volleyball players, which are essential in actual situations on a volleyball court. In our opinion, this strategy is the results of the players’ long training and thus adoption of certain habits. In volleyball training great importance is attached to the players’ ready position, which on the one hand should be stable, but on the other hand should provide the convenience of a quick reaction to an incoming ball in any direction. During a volleyball game, players must always be ready to take action, primarily related to a quick movement. Therefore, these mean velocity values may be due to volleyball players’ habit of showing constant readiness. A higher mean velocity was also noted in volleyball players by Agostini et al. [1], who studied mainly players on lower sporting levels. Like in the present study, it is justified by the volleyball players’ habitual readiness to act and the need to react quickly. The question remains, however, why the better trained female volleyball players displayed a lower mean velocity than their less advanced male counterparts. We believe there is a transition phase in the process of adjustment of postural strategies in second-division male volleyball players.

The elite female players achieved slightly higher frequency values than the control group, although - apart from peak frequency (PF) on the coronal plane - these values were not statistically significant. Similar results were reported by Wulf [25] in her study of elite balance acrobats. She believes that they can be an indication of an external focus of attention. Following the constrained-action hypothesis [5], attempts at controlling one’s own movements constrain the motor system by interfering with automatic control processes that would normally regulate the movement [5, 26–28]. The participants who focused only on their own feet (internal focus of attention) controlled the balance less effectively than those who focused on the movement effect (external focus of attention). Volleyball players aim to score a point either by winning a rally through a successful play or by making the opposing team commit a fault. The way to achieve this goal (sequence of movements), and certainly muscle control, are automatic actions. Conscious muscle tone control or sequences of movements in offensive or defensive play would interfere with achieving the goal of scoring. Higher frequency values demonstrate a better level of automatism of balance control. On the other hand, high postural sway frequency values are considered to involve a higher energy expenditure. Such results have been observed in older people. We therefore believe that not high, but optimal frequency values are best for correct, but at the same time, economical postural control. According to Kuczyński et al. [19], higher frequency values may mean that automatism in postural sway control requires increased nervous system activity. The transition from conscious to automatic control is accompanied by increased posture monitoring. This may be an integral part of automatic posture control to compensate for less attention, or a precaution against unexpected risks. Wulf et al. [29] also believe that focusing on the movement effect, rather than on the movement itself, optimizes muscle coordination. Increased efficiency and economy of movement is related to the sporting advancement level. When movement is automated through training, it is performed more efficiently (e.g. with less neuromuscular activity) [29]. These results correspond perfectly with ours. The players of the Poland national women’s volleyball team achieved slightly higher frequency values than the controls, but lower than the second-league male volleyball players [14]. In our opinion, the players at the lower sports level are in a transitional phase of adjusting their postural strategies. Their balance control remains at a high level, however, in terms of energy expenditure they perform worse than better trained athletes. To sum up, we believe that top-class female volleyball players follow a specific postural control strategy, which provides them not only with the readiness and ability to react quickly in any direction, but also with excellent economy of movement.

In our study we observed a higher COP fractal dimension of the Poland national women’s volleyball team players as compared with controls. It is assumed that low values of this parameter indicate better stability, while its high values demonstrate better adaptability. This provides further evidence that the strategy of maintaining balance by top-level athletes is highly flexible. It shows the use of more degrees of freedom [30]. This is the result of their extensive sensorimotor “experience”, which has contributed to the development of available motion programs. Moreover, a high fractal dimension is attributed to a better use of sensory signals [31, 32]. When the situation on the court requires it, volleyball players adopt a stable posture to perform their tasks precisely. However, they also display excellent adaptability to changing conditions during the game and to the need to act quickly. As a result, the elite volleyball players have the capacity to optimally select postural strategies for the situation during a game. On the other hand, volleyball players representing a lower level of sports advancement (second league) have even higher values of this parameter [14]. The very high fractal dimension means that they are still striving for such an optimal strategy.

In conclusion, it can be assumed that due to the high motor requirements of their sport discipline Polish female volleyball players have developed a unique posture control. On the court they have to distribute their sensory resources optimally between balance control and actions resulting from the specifics of the volleyball game. Thus, due to the inevitable trade-off between stability and maneuverability [33, 34], exceptional postural control is a prerequisite for volleyball players. Most of the actions on the court have a ‘destabilization – recovery of balance’ sequence that boost demands not only on spatial but also on temporal balance abilities [35]. The static stability is continuously challenged by the demand for an optimal body position prior to actions with the ball. Thus, the postural sway variability should be limited to ensure precision and efficiency in action, but should also provide the CNS with adequate exploratory capabilities to cope with changing situation on the court. Somewhat different balance skills are crucial in ensuring good stability in approaching and landing for spiking or blocking jumps and also in controlling body posture in aerial phases of spiking [36].

Our study has some limitations. The sample size (*N* = 31 for both groups) is sensitive enough to detect an r effect size 0.3 or larger only, with 80% of power, and 5% significance level. It is a medium effect size and our results must be interpreted accordingly. Small differences cannot be detected with that study design, and thus some problems can be found (e.g., sampling error as a source of bias, etc.). However, it should be remembered that this is a unique research material comprising a group of carefully selected two-time European volleyball champions. Another limitation of the present study is the players’ performance of only one quiet standing task with their eyes open. Future studies comparing top-level players and controls performing several different standing tasks will provide more information on other aspects of top-ranking volleyball players’ postural control. Finally, one can be tempted to speculate that the between-group differences in age, height and body mass might have affected the final results. This however seems implausible since there are no studies which revealed the effect of the latter variables on postural control in young healthy persons.

## Conclusions

Elite female volleyball players developed a unique posture control based on a compromise between stability and maneuverability. In particular, they demonstrated more robust stability which is less vulnerable to accidental fluctuations and unexpected situations on court. They significantly advanced the ability to use external focus of attention. Their balance regulation is highly flexible with broad spectrum of postural strategies promoting the capacity to optimally select these strategies according to the demands of game.

## Data Availability

The datasets used and/or analyzed during the current study are available from the corresponding author on reasonable request.

## Abbreviations

COP: Center of pressure
COM: Center of mass
ML: The medial-lateral plane
AP: Anterior-posterior plane
SD: Standard deviation
RA: Range
MV: Mean velocity
PF: Peak frequency of COP-COM correction signal
CF: COP frequency based on normalized path COP length
FD: Fractal dimension

## References

[1] Agostini V, Chiaramello E, Canavese L, Bredariol C, Knaflitz M (2013). Postural sway in volleyball players. Hum Mov Sci.

[2] Alpini D, Mattei V, Schlecht H, Kohen-Raz R (2008). Postural control modifications induced by synchronized ice skating. Sport Sci Health.

[3] Kuczyński M, Szymańska M, Bieć E (2011). Dual-task effect on postural control in high-level competitive dancers. J Sports Sci.

[4] Stambolieva K, Diafas V, Bachev V, Christova L, Gatev P (2012). Postural stability of canoeing and kayaking young male athletes during quiet stance. Eur J Appl Physiol.

[5] Wulf G (2013). Attentional focus and motor learning: a review of 15 years. Int Rev Sport Exerc Psychol.

[6] Ihalainen S, Linnamo V, Mononen K, Kuitunen S (2016). Relation of elite rifle shooters’ technique-test measures to competition performance. Int J Sports Physiol Perform.

[7] Era P, Konttinen N, Mehto P, Saarela P, Lyytinen H (1996). Postural stability and skilled performance-a study on top-level and naive rifle shooters. J Biomech.

[8] Paillard T, Noé F, Rivière T, Marion V, Montoya R, Dupui P (2006). Postural performance and strategy in the unipedal stance of soccer players at different levels of competition. J Athl Train.

[9] Asseman F, Caron O, Crémieux J (2005). Effects of the removal of vision on body sway during different postures in elite gymnasts. Int J Sports Med.

[10] Asseman F, Caron O, Crémieux J (2004). Is there a transfer of postural ability from specific to unspecific postures in elite gymnasts?. Neurosci Lett.

[11] Sell TC, Tsai Y-S, Smoliga JM, Myers JB, Lephart SM (2007). Strength, flexibility, and balance characteristics of highly proficient golfers. J Strength Cond Res.

[12] Kim M, Kim Y, Kim H, Yoon B (2018). Specific muscle synergies in national elite female ice hockey players in response to unexpected external perturbation. J Sports Sci.

[13] Gautier G, Thouvarecq R, Vuillerme N (2008). Postural control and perceptive configuration: influence of expertise in gymnastics. Gait Posture..

[14] Kuczyński M, Rektor Z, Borzucka D. Postural control in quiet stance in the second league male volleyball players. Hum Mov. 2009;10. 10.2478/v10038-008-0025-4.

[15] Welch TDJ, Ting LH (2014). Mechanisms of motor adaptation in reactive balance control. PLoS One.

[16] Söderman K, Werner S, Pietilä T, Engström B, Alfredson H (2000). Balance board training: prevention of traumatic injuries of the lower extremities in female soccer players?. Knee Surgery, Sport Traumatol Arthrosc.

[17] Verhagen E, van der Beek A, Twisk J, Bouter L, Bahr R, van Mechelen W (2004). The effect of a proprioceptive balance board training program for the prevention of ankle sprains. Am J Sports Med.

[18] McGuine TA, Keene JS (2006). The effect of a balance training program on the risk of ankle sprains in high school athletes. Am J Sports Med.

[19] Prieto TE, Myklebust JB, Hoffmann RG, Lovett EG, Myklebust BM (1996). Measures of postural steadiness: differences between healthy young and elderly adults. IEEE Trans Biomed Eng.

[20] Kuczyński M (1999). The second order autoregressive model in the evaluation of postural stability. Gait Posture..

[21] Mangiafico SS (2016). R handbook: two-sample Mann–Whitney U test.

[22] Horak FB (2006). Postural orientation and equilibrium: what do we need to know about neural control of balance to prevent falls?. Age Ageing.

[23] Haddad JM, Rietdyk S, Claxton LJ, Huber JE (2013). Task-dependent postural control throughout the lifespan. Exerc Sport Sci Rev.

[24] Paillard T. Relationship between sport expertise and postural skills. Front Psychol. 2019;10. 10.3389/fpsyg.2019.01428. 10.3389/fpsyg.2019.01428 PMC6603331 31293483

[25] Wulf G (2008). Attentional focus effects in balance acrobats. Res Q Exerc Sport.

[26] Wulf G, McNevin N, Shea CH (2001). The automaticity of complex motor skill learning as a function of attentional focus. Q J Exp Psychol A.

[27] Wulf G, Shea C, Park J-H (2001). Attention and motor performance: preferences for and advantages of an external focus. Res Q Exerc Sport.

[28] Wulf G, Mercer J, McNevin N, Guadagnoli MA (2004). Reciprocal influences of Attentional focus on postural and Suprapostural task performance. J Mot Behav.

[29] Wulf G, Shea C, Lewthwaite R (2010). Motor skill learning and performance: a review of influential factors. Med Educ.

[30] Sternad D (2018). It’s not (only) the mean that matters: variability, noise and exploration in skill learning. Curr Opin Behav Sci.

[31] Casabona A, Leonardi G, Aimola E, La Grua G, Polizzi CM, Cioni M (2016). Specificity of foot configuration during bipedal stance in ballet dancers. Gait Posture..

[32] Cone BL, Goble DJ, Rhea CK (2017). Relationship between changes in vestibular sensory reweighting and postural control complexity. Exp Brain Res.

[33] Huang HJ, Ahmed AA (2011). Tradeoff between stability and maneuverability during whole-body movements. PLoS One.

[34] Acasio J, Wu M, Fey NP, Gordon KE (2017). Stability-maneuverability trade-offs during lateral steps. Gait Posture.

[35] Le Mouel C, Brette R. Mobility as the purpose of postural control. Front Comput Neurosci. 2017;11. 10.3389/fncom.2017.00067. 10.3389/fncom.2017.00067 PMC5529402 28798679

[36] Pau M, Kim S, Nussbaum MA (2012). Does load carriage differentially alter postural sway in overweight vs. normal-weight schoolchildren?. Gait Posture..

